# Predicting Determinants for Conversion of Off-Pump Coronary Revascularization to On-Pump Surgery: A Retrospective Analysis

**DOI:** 10.7759/cureus.42258

**Published:** 2023-07-21

**Authors:** Chandranshu Kumar, Sridartha K, Rahul Bhushan, Vijay Grover, Narender S Jhajhria, Palash V Aiyer

**Affiliations:** 1 Cardiothoracic Surgery, Dr. Ram Manohar Lohia Hospital, New Delhi, IND

**Keywords:** coronary artery bypass grafting (cabg), cabg surgery, off-pump to on-pump conversion, off-pump coronary artery bypass grafting (opcabg), adult coronary surgery on and off pump

## Abstract

A global consensus has not yet been reached regarding the preference for off-pump versus on-pump coronary revascularization. Although the coronary trial indicates that the secondary endpoint outcomes favor on-pump surgery, the cost-effectiveness and significantly lower immediate and early complications in off-pump surgery make it favorable for the Indian population. To analyze patients who underwent coronary revascularization, specifically coronary artery bypass grafting (CABG), a retrospective five-year study was conducted. During the given duration, a total of 652 patients underwent CABG. The study revealed a positive correlation between diabetes, high body surface area (BSA), and preexisting renal dysfunction as strong predictors for converting off-pump coronary artery bypass surgery (OPCABG) to on-pump surgery coronary artery bypass surgery (ONCABG). Preoperative electrocardiographic (ECG) changes and the use of intra-aortic balloon pulsation (IABP) as a mechanical assist device were strongly associated with the incidence of conversion from OPCABG to ONCABG. Tight left main disease and ostial coronary disease indicate a progressive dysfunction during off-pump surgery, necessitating early conversion to on-pump surgery to avoid complications. The on-pump group had more adverse outcomes in regard to renal and neurological dysfunction, which can be attributed to pump-induced dysfunction. In such scenarios, a surgeon's preparedness to convert an OPCABG to an ONCABG can be swift and efficient. In anticipation of increased pump-related complications in the ONCABG group, a measured approach can be implemented to avoid adverse postoperative outcomes in high-risk patients.

## Introduction

In 1967, Kolessov reported the first case series of coronary artery bypass grafting (CABG) performed using the off-pump technique on a beating heart [1]. Off-pump CABG (OPCAB) gained momentum in the late nineties due to its evident advantages over on-pump CABG (ONCAB). OPCAB has proven to be cost-effective and associated with a lower incidence of coagulopathy, inflammatory response, neurological impact, hospital stays, and adverse postoperative outcomes [2]. It also requires less use of ionotropic agents, results in reduced postoperative bleeding, and lower rates of renal dysfunction compared to ONCAB surgery [3]. However, despite these advantages, ONCABG gained traction in subsequent years due to easier surgical access, complete grafting, better long-term survival rates, and a lesser rate of revascularization [4]. There is still no global consensus regarding the preference for off-pump vs. on-pump coronary revascularization. While the secondary endpoint outcomes favor on-pump surgery, as indicated by the coronary trial, the cost-effectiveness and significantly lower immediate and early complications of off-pump surgery make it favorable in the Indian population [5]. Switching from OPCAB to on-pump surgery is considered adverse and is reserved for situations with unfavorable intraoperative hemodynamics [6]. In our study, we aimed to identify preoperative underlying risk factors that can serve as predictors for the need to convert to on-pump surgery during routine off-pump coronary revascularization. We also aimed to compare the short-term and midterm outcomes of patients who underwent coronary revascularization in both groups.

## Materials and methods

A retrospective study was conducted to analyze patients who underwent coronary revascularization, specifically coronary artery bypass grafting (CABG), at our tertiary care center at ABVIMS and Dr RML Hospital in New Delhi from May 2017 to May 2022. Data analysis included presenting features, history of infarction, electrocardiographic (ECG) findings, echocardiographic (ECHO) findings, disease severity, coronary anatomy in coronary angiogram, presence of comorbidities, glycemic control, the need for intra-aortic balloon assist device, and postoperative outcomes in all patients.

The preferred surgical approach for CABG at our center is the off-pump approach. The decision to convert an off-pump CABG to on-pump was based on clinical and diagnostic findings suggestive of overt left ventricular dysfunction. Indicators used for the decision included elevated pulmonary artery (PA) pressures, hemodynamic instability refractory to ionotropic agents, increased airway pressure, transesophageal echo (TEE) findings indicating moderate to severe myocardial dysfunction and regional wall motion abnormalities, severe arrhythmias, and heart distension with poor contractility. The presence of any one or more of these findings was considered an indication to switch to on-pump CABG. Differentiation between on-pump beating heart and on-pump arrested heart was not performed in this study. Patients with comorbidities such as severe mitral regurgitation, LV aneurysm/pseudoaneurysm, concurrent valvular procedures, post-infarction ventricular septal defects, and other conditions where on-pump surgery was indicated preoperatively were not included in our study. The use of the intra-aortic balloon pulsation (IABP) assist device was based on preoperative hemodynamic instability, fresh ECG changes, tight left main disease, and severe left ventricular dysfunction.

A standard surgical technique of midline sternotomy was employed for all patients. The left internal mammary artery (LIMA) was harvested using a no-touch technique and used as the standard graft for the left anterior descending artery (LAD) coronary artery in all cases. Following LIMA harvest, pericardiotomy and systemic heparinization (200 IU/Kg) were performed. The Medtronic Octopus stabilizer was used, and grafting to the LAD was completed using 7-0 prolene sutures. A left radial graft or great saphenous vein graft to other distal coronaries was performed in accordance with standard protocols and recommendations using 7-0 prolene sutures. If subsequent ventricular dysfunction, as discussed earlier, became evident at any stage, the procedure was paused, and complete institution of cardiopulmonary bypass (CPB) was performed. The remaining grafting in such cases was conducted on the beating heart with CPB. Subsequently, the aorta was partially clamped using a Derra clamp, and proximal anastomosis was completed using 5-0 prolene sutures for vein grafts and 6-0 prolene sutures for radial grafts. Heparin was completely reversed with protamine in all cases, and the incision was closed in layers after achieving adequate hemostasis. Routine follow-up was conducted with weekly visits for six weeks, followed by monthly visits for six months, and subsequently, half-yearly follow-up visits were performed for all patients.

## Results

A total of 652 patients were operated for CABG in the five-year duration of our study on data review in the given period. In total, 534 patients underwent CABG with beating heart off-pump surgery (82%), while 118 patients (18%) needed a conversion to on-pump surgery as per intraoperative requirements in accordance with the criteria mentioned in Table 1.

**Table 1:** Comparative data between off-pump and on-pump group IABP = intra-aortic balloon pulsation; ECG = electrocardiogram; ECHO = echocardiograph; LV = left ventricle; MR = mitral regurgitation; RWMA = regional wall motion abnormality; LVEDD = left ventricular end-diastolic diameter; CAG = coronary angiogram; TVD = triple vessel diease

	Off-pump (non-conversion) n (%)	On-pump (conversion) n (%)
Total number of patients	534	118
Age >65 years	118 (22%)	10 (8.4%)
Male/female ratio	442/92 (4.8:1)	93/25 (3.7:1)
Body surface area>1.7	97 (18.1%)	31 (26.2%)
Hypertensive	200 (37.4%)	31 (26.2%)
Diabetic	167 (31.2%)	41 (34.7%)
Active smoker	76 (14.2%)	9 (7.6%)
Renal dysfunction	6 (1.1%)	8 (6.7%)
IABP used preoperatively	46 (8.6%)	18 (15.2%)
Recent history of Infarction	193 (36.1%)	37 (31.3%)
Acute ECG changes	23 (4.3%)	19 (16.1%)
ECHO (LV ejection fraction<35%)	34 (6.3%)	12 (10.1%)
ECHO (Moderate MR, presence of moderate-severe RWMA)	36 (6.7%)	25 (21.1%)
LVEDD> 5 centimeters	24 (4.4%)	21 (17.8%)
CAG (LM disease)	26 (4.8%)	18 (15.2%)
CAG (Ostial TVD with lesions>90%)	87 (16.3%)	41 (34.7%)

Comparing the results of the two groups from the table above, the following findings were derived: Elderly population and sex were not significant determinants for conversion to an on-pump procedure in our study, as indicated by the lack of positive predictive value on multivariate analysis.

A higher body surface area (BSA) was associated with an increased predisposition to conversion to an on-pump procedure. The on-pump group had 26.2% of patients with a higher BSA, compared to 18.1% in the off-pump group.

Systemic preexisting hypertension did not show a positive predictive value in our study, while diabetics were mildly more susceptible to conversion. The on-pump group had a comparative data of 34.7% diabetic population, while the off-pump group had 31.2%.

Preexisting renal dysfunction with elevated creatinine levels was found to be more susceptible to conversion. Our data showed that 6.7% of patients in the on-pump group were affected, compared to 1.1% in the off-pump group. The preoperative use of intra-aortic balloon pulsation (IABP) was more common in the on-pump group (15.2%) than in the off-pump group (8.6%). This correlation supports the understanding that acute left ventricular (LV) dysfunction with fresh ECG changes is more likely to be converted to an on-pump procedure.

History of recent myocardial infarction (within 30 days of surgery) did not show a positive correlation with conversion to an on-pump procedure. Acute ECG changes were a strong predictor for conversion from an off-pump procedure to an on-pump, with a comparative value of almost 4:1 in favor of the on-pump group. Preexisting left ventricular dysfunction was slightly higher in the on-pump group (10.1%) compared to the off-pump group (6.3%).

The incidence of preoperative mitral regurgitation and regional wall motion abnormalities (RWMA) was higher in the on-pump group (21.1%) vs. the off-pump group (6.7%). LV dilatation on preoperative echocardiography showed a strong positive correlation with the on-pump group, with an incidence rate of 17.8% compared to 4.4% in the off-pump group. Left main disease and tight ostial lesions also showed a strong positive correlation with the on-pump group compared to the off-pump group (Tables 2, 3, 4).

**Table 2:** Complications OPCABG = off-pump coronary artery bypass grafting; ONCABG = on-pump coronary artery bypass grafting; LV = left ventricle; ECHO = echocardiogram

Early complications	OPCABG n (%)	ONCABG n (%)
Exploration for bleeding	22 (4.1%)	12 (10.1%)
Arrhythmia	24 (4.5%)	9 (7.6%)
Wound infection	38 (7.1%)	10 (8.4%)
Neurological complications	17 (3.1%)	8 (6.7%)
Renal dysfunction	14 (2.6%)	5 (4.2%)
Intra operative Infarction	16 (3%)	9 (7.6%)
LV dysfunction on ECHO	37 (7%)	13 (11%)
Early deaths	19 (3.5%)	7 (6%)

**Table 3:** Chi-squared test OPCABG = off-pump coronary artery bypass grafting; ONCABG = on-pump coronary artery bypass grafting; CKD = chronic kidney disease; BSA = body surface area

	OPCABG_AGE	ONCABG_AGE	OPCABG _SEX	ONCABG _SEX	OPCABG _DIABETES	ONCABG _DIABETES	OPCABG _HYPERTENSION	ONCABG _HYPERTENSION	ONCABG _CKD	OPCABG _CKD	ONCABG _BSA	OPCABG _BSA
Chi-squared	416.831^a^	49.610^b^	230.282^c^	39.186^d^	75.516^c^	10.983^d^	34.065^c^	26.576^d^	510.270^e^	88.169^d^	5.904E3^a^	54.271^b^
df	42	28	1	1	1	1	1	1	1	1	48	43
Sig. value	0	0.007	0	0	0	0.001	0	0	0	0	0	0.116

**Table 4:** Descriptive statistics on data BSA = body surface area; LVEDD = left ventricular end-diastolic dimension; ONCABG = on-pump coronary artery bypass; OPCABG = off-pump coronary artery bypass

	N	Mean	Std. deviation	Minimum	Maximum
ONCABG BSA	535	2.1246	8.81051	1	180
OPCABG BSA	118	1.6161	0.13105	1.36	1.91
ONCABG LVEDD	534	4.1878	1.79117	0.4	43
OPCABG LVEDD	118	4.4992	0.77011	3	6.8

Re-exploration for bleeding was observed more frequently in the on-pump group, possibly due to a higher dose of heparinization, pump-induced hemolysis, and coagulopathy. The higher incidence of suture lines for venous and aortic cannulation sites in this group may also contribute to the increased need for re-exploration.

The incidence of arrhythmias and wound infections was similar in both subgroups, with no statistical significance. Neurological complications and renal dysfunction in the early postoperative period were significantly more common (almost twice as high) in the ONCABG group compared to the OPCABG group.

The incidence of fresh infarction observed on ECG and early left ventricular dysfunction were also higher in the ONCABG group, likely due to compromised heart function and the occurrence of adverse events that led to the conversion to on-pump surgery. The early mortality rate in the off-pump subgroup was 3.5%, compared to 6% in the on-pump group.

There was a mean loss of follow-up of 16.2% in both subgroups during the midterm follow-up. Routine follow-up echocardiography assessments were performed every six months for both subgroups of patients. In the OPCABG group, 118 patients (22%) showed improvement in left ventricular function and regional wall motion abnormalities (RWMA), while 225 patients (42.1%) showed no change in left ventricular function. A total of 12.1% of patients in the same group experienced a decline in left ventricular function during the midterm follow-up. Additionally, 43 patients (8%) reported a progression of symptoms from The New York Heart Association (NYHA) class 2 to class 3 in the OPCABG group, but these cases were successfully managed by adding diuretics and optimizing medical therapy. None of the patients in either group required repeat revascularization. All severely symptomatic patients with severe left ventricular dysfunction on echocardiography underwent a review angiogram, and all patients had patent left internal mammary artery (LIMA) grafts.

In the ONCABG group, during the midterm follow-up, 30 patients (25.4%) showed improvement in left ventricular function and RWMA, while 53 patients (45%) showed no change in the echocardiography evaluation. A total of 15% of patients experienced a decline in left ventricular function, and 12% of patients showed progression of symptoms from NYHA class 2 to class 3 during the postoperative course. All of these patients were successfully managed medically with appropriate drug therapy.

## Discussion

OPCAB is a preferred modality for coronary revascularization in India due to its lower incidence of complications and the financial constraints of an economically developing nation. With surgical expertise and careful handling of the heart using stabilizers and optimal intraoperative use of inotropes, the limitations of OPCABG can be overcome, making it an attractive alternative to ONCABG [7]. We successfully implemented a strategy of performing OPCABG surgery as the preferred modality at our center, reserving conversion to on-pump surgery for adverse events and as a surgical backup option during challenging situations.

Thorough preoperative evaluation can serve as a predictor for adverse intraoperative and postoperative events, enabling a carefully tailored approach for better preparedness and resource optimization.

A positive correlation was observed in diabetics, patients with a high body surface area (BSA), and patients with preexisting renal dysfunction as strong predictors for the conversion from an off-pump procedure to an on-pump surgery. Implementing an aggressive weight loss regimen, optimizing glycemic control, and optimizing kidney function while avoiding nephrotoxic drugs can help mitigate the negative impact of these comorbidities on cardiac function [8,9].

Preoperative ECG changes and the use of the intra-aortic balloon pump (IABP) as a mechanical assist device were strongly associated with the incidence of conversion from OPCABG to ONCABG. This underscores the fact that progressive myocardial dysfunction in the immediate preoperative period is associated with adverse outcomes and often requires on-pump support. These hearts also have less tolerance for the handling and maneuverability needed for distal grafting, making preparedness for on-pump surgery a high priority for surgeons [10,11].

Likewise, tight left main disease and ostial coronary disease indicate an impending dysfunction that can progress during an off-pump surgery, necessitating early conversion to on-pump surgery to prevent progressive left ventricular dysfunction.

The on-pump group experienced more adverse outcomes in terms of renal and neurological dysfunction, which can be attributed to pump-induced complications. Adopting a neuroprotective approach, using filtration during on-pump procedures, maintaining an adequate flow rate, and aiming for early extubation should be prioritized in ONCABG patients to minimize these complications [12,13].

Early initiation of beta-blockers or the use of amiodarone to prevent arrhythmias should also be a priority in the ONCABG group, considering the higher incidence of postoperative arrhythmias observed in this group [14].

## Conclusions

In conclusion, our study reinforces the preference for off-pump coronary artery bypass grafting (OPCABG) as a favorable modality for coronary revascularization, especially in the Indian population. OPCABG offers several advantages, including reduced immediate and early complications, cost-effectiveness, and shorter hospital stays. Our findings highlight the importance of careful patient selection and preoperative evaluation to identify predictors for the need for conversion from off-pump to on-pump surgery. 

While off-pump surgery presents numerous benefits, the decision to convert to on-pump surgery should be made judiciously, considering adverse events and unfavorable intraoperative conditions. The on-pump group demonstrated a higher incidence of adverse outcomes, particularly in terms of renal and neurological complications, which can be attributed to pump-induced dysfunction. To mitigate these risks, implementing neuroprotective strategies, using filtration during on-pump procedures, maintaining adequate flow rates, and early extubation should be prioritized.

Overall, our study highlights the importance of individualized patient management, careful consideration of risk factors, and a tailored surgical approach in optimizing outcomes for coronary revascularization procedures. Further research and larger-scale studies are warranted to strengthen the evidence base and establish a global consensus on the preference for off-pump versus on-pump coronary revascularization.
